# Community pharmacy resiliency during Covid-19 pandemic in Iran: A qualitative study

**DOI:** 10.1016/j.rcsop.2025.100670

**Published:** 2025-10-03

**Authors:** Zhivan Zomorodi, Faezeh Valaei Sharif, Najmeh Moradi, Zahra Sharif

**Affiliations:** a Faculty of Pharmacy, Alborz University of Medical Sciences, Karaj, Iran; b Health Economics Group, Population Health Sciences Institute, Baddiley-Clark Building, Newcastle University, Newcastle upon Tyne NE2 4BN, UK

**Keywords:** COVID-19, Resiliency, Resilient healthcare, Pharmacies, Pandemic preparedness, Iran

## Abstract

**Background:**

The COVID-19 pandemic has profoundly impacted global healthcare systems. This qualitative study explores how community pharmacists in Iran demonstrated resilience during this disaster. Despite challenges like medication shortages, Personal Protective Equipment deficiencies, and staffing issues, pharmacists implemented innovative measures such as social distancing protocols, remote consultations, and home delivery services. Insights from this study inform strategies to enhance healthcare system preparedness for future public health preparedness.

**Objective:**

This study qualitatively explores experiences of Iranian pharmacists during the COVID-19 pandemic, focusing on resilience, emergency management strategies, challenges faced and future emergency preparedness.

**Methods:**

Semi-structured interviews were conducted with 25 pharmacists (14 men, 11 women; mean practice experience: 10–14 years) in Tehran and Alborz provinces. Data were analyzed thematically using MAXQDA and following Braun and Clarke's six-phase framework*.*

**Results:**

Analysis of 1260 codes and 11 categories revealed that pharmacists faced operational challenges, including increased work load, psychological strain, financial constraints, and supply shortages. In response, they adapted creatively, employing strategies such as teleconsultations, public health education, inventory sharing, and strict safety protocols. However, participants consistently highlighted policy and systemic gaps, including insufficient governmental support and the limited integration of pharmacists into crisis management frameworks.

**Conclusion:**

Community pharmacies played a critical role during the COVID-19 pandemic by adapting rapidly and expanding public health services. Strengthening telepharmacy infrastructure, supply chain policies, and integrating pharmacists into national preparedness frameworks is essential to improve healthcare system resilience in future healthcare emergencies*.*

## Highlights

• Pharmacies in Iran faced staff, supply, financial, and communication challenges.
• Resilience strategies included task shifts, rationing, and remote patient counseling.
• Later waves saw better procurement, education, and expanded disease management.
• Policies should recognize pharmacists' roles and ensure early guideline updates.

## 1 Introduction

The profound global repercussions of the COVID-19 pandemic, stemming from the novel coronavirus SARS-CoV-2, have been extensively documented.^1^, ^2^, ^3^ Originating in late 2019 in China, the virus rapidly disseminated, resulting in millions of confirmed cases and hundreds of thousands of deaths worldwide.^4^ As part of the effort to control the outbreak, the majority of countries adopted non-pharmacological behavioral interventions (NPIs) including hand hygiene techniques, environmental hygiene, social distancing, closing non-essential businesses, schools, and universities, implementing travel restrictions, quarantining, and even lockdown.^5^^,^^6^ On March 11, 2020, the World Health Organization (WHO) declared COVID-19 a global pandemic. Iran reported its initial confirmed cases on February 19, 2020.^7^ Despite some countries' efforts to eradicate the novel virus, most countries assumed widespread community transmission was inevitable. Regardless of the approach, a clear and timely response strategy would empower the governments to develop or adjust emergency legislation and planning.^8^ Iran established the “COVID-19 Epidemiology Committee” on March 28, 2020, to examine epidemiological conditions, review documents and facts, and predict disease trends based on hypothetical scenarios including physical/social distancing, isolation, quarantine, and lockdown.^9^ The capital city of Iran, Tehran, faced five consecutive waves of the disease. The first COVID-19 epidemic wave began on 01/03/2020 and lasted 51 days. It was followed nearly 3 months later by the second epidemic wave, which started on 04/06/2020 and lasted 23 days. The second wave was as a resulte of eased restrictions and declining social distancing measures, such as holding ceremonies.^10^^,^^11^ Even though restrictions such as social distancing resulted in significant economic impacts, including declining stock markets, increased government spending, and unemployment, the mortality benefits may have been greater during the COVID-19 pandemic.^12^

During disruptions such as pandemics, a health system's ability to “improve, maintain, or restore health” can be significantly compromised, as seen in this pandemic, which has severely impacted public health systems worldwide, leading to a surge in cases and deaths while hindering the provision of basic healthcare services.^13^^,^^14^ The impact on the healthcare system has been particularly substantial in regions with limited infrastructure. Hospitals and clinics adapted infection prevention protocols, incorporating Personal Protective Equipment (PPE) and modifying facilities. Healthcare workers' infections exacerbated staff shortages that intensified the strain on the system.^15^ Similar indirect effects of the pandemic have also been documented in other health systems, where service disruptions, staff strain, and resource shortages further highlighted systemic vulnerabilities and the need for resilient preparedness strategies.^16^

Pharmacists, in collaboration with other healthcare professionals, played a pivotal role in addressing COVID-19 challenges from its onset, in mitigating community transmission. Their responsibilities extended beyond medications, preventive products, and medical supplies. Pharmacists also disseminated vital health information regarding the disease and contributed to early detection efforts. Community pharmacies and their teams, serving as essential healthcare providers and direct access points, maintained their operations during the pandemic despite facing challenges such as medication shortages, deficiencies in PPE, and staffing issues. Additionally, social distancing measures required significant adjustments to protocols for medication dispensing and, for some, difficulties sustaining services throughout the ongoing crisis.^5^^,^^6^^,^^17^^,^^18^ Similar patterns have been reported globally, where community pharmacists assumed expanded frontline roles to maintain patient care and public health support during the pandemic. For example, pharmacists adopted telepharmacy, home delivery services, balanced supply chains, delivered accurate public health messaging, provided remote consultations, and educated patients to maintain adherence to their current treatment regimens.^17^^,^^19^

In the face of a crisis like the COVID-19 pandemic, organizational capabilities are crucial for maintaining the same function, structure, and characteristics. These capabilities, often referred to as resilience, enable organizations to navigate challenging circumstances effectively. Resilience, in the organizational context, is defined as “the ability of a system to absorb disturbances and reorganize while undergoing change.” A resilient social system can absorb temporary or permanent risks and adapt to rapidly changing conditions without losing its functionality.^20^ The ability of societal organizations to manage crises and challenges significantly influences a society's capacity to face such adversities and recover effectively. Organizations, through the critical services they provide during crises, play a pivotal role in expediting society's return to normal conditions.^21^ A resilient health system is vital for the attainment of Universal Health Coverage (UHC) and health security by enabling continued service delivery during crises. UHC facilitates access to essential healthcare services without imposing financial burdens, while health security aims to mitigate the effects of public health emergencies. Both objectives necessitate robust, integrated health systems to provide equitable and high-quality healthcare across populations.^22^ Supporting this background, this study aimed to qualitatively explore the experiences of pharmacists in community pharmacies in managing the COVID-19 pandemic crisis in Iran, identify factors leading to resiliency, and assess their levels of resiliency and adaptability. Despite these global insights, the Iranian context remains underexplored. By highlighting both strengths and systemic gaps, the findings of this study offer valuable lessons to inform policy development and improve health system preparedness for future pandemics and public health emergencies.

## 2 Method

This study adhered to the Consolidated Criteria for Reporting Qualitative Research (COREQ).^23^ The completed checklist is provided in Supplementary File 1.

### 2.1 Research team and reflexivity

The interviews were conducted by JZ and FV. JZ and FV were female PharmD students. Both received prior formal training in semi-structured interviewing and qualitative research from two experienced female faculty members (ZS, NM) who hold PhDs in Pharmacoeconomics. There was no prior relationship between interviewers and participants before the study. Participants were informed of the interviewer's academic background and the study's aim to explore pharmacy resilience during the COVID-19 pandemic. Interviewers acknowledged their professional background in pharmacy and remained neutral to reduce bias during data collection. The interviewers discussed potential personal biases during regular team meetings to minimize their influence on data interpretation.

### 2.2 Study design

This research employed a descriptive qualitative approach to investigate the experiences of community pharmacies of Tehran and Alborz provinces in managing the COVID-19 pandemic and was carried out in 2021. The notion of the research question guided the study, and a semi-structured interview approach was utilized. The interviews began with specific questions and allowed for additional inquiries that emerged during the interview, forming the foundation of the semi-structured interview process.^24^ A qualitative approach was chosen to gain an in-depth understanding of pharmacists' lived experiences, coping mechanisms, and decision-making processes in a complex, rapidly evolving public health crisis.^25^

### 2.3 Setting and participants

In this qualitative study, a purposive sampling strategy was employed to recruit participants who could provide rich insights into pharmacy resilience during the pandemic. Participants were recruited via professional networks and pharmacy associations using direct outreach through email, phone calls, and in-person visits. The sample was selected based on the research context and study entry criteria. The target population comprised pharmacists working in pharmacies during COVID-19 pandemic in Tehran and Alborz provinces, two of Iran's most populated regions. Inclusion criteria specified being a pharmacist or a pharmacy owner, having a minimum of 3 years of experience in community pharmacy, actively working in a community pharmacy for a minimum of 24 h per week in the past three years, and working in pharmacies during COVID-19 pandemic for at least six months in Tehran and Alborz provinces. No eligible participants declined to participate or dropped out during the study.

### 2.4 Data collection and analysis

To ensure the depth and relevance of the questions, a set was drafted after reviewing pertinent literature on pharmacy resilience, adaptability, and experiences in other countries. The guide included six predefined open-ended questions exploring pharmacies' experiences during the first and second waves of COVID-19 in Iran, changes in routine activities and provision of pharmaceutical and non-pharmaceutical services COVID-19, difficulties encountered during different pandemic waves, and solutions employed to overcome these challenges. The first wave of COVID-19 began in March 2020 and the second wave began in the middle of summer 2020. Both waves lasted about three to four months. Overall, there were six predefined questions that directed the interviews and other questions emerged based on the flow of the interviews. A semi-structured interview guide (Supplementary File 2) was used to ensure consistency across interviews. No repeat interviews were conducted. The time and place of interviews were scheduled according to participants' preferences. Interviews were conducted in Persian, either face-to-face in private consultation spaces, or online via secure messaging platforms with audio file exchanges. Interviews lasted for 20 to 40 min and adhered to the same set of predetermined questions, with pharmacists encouraged to express additional opinions. Field notes were taken after each session to capture non-verbal cues and contextual observations and were maintained to ensure transparency and traceability during the analytic process. Participants' consent was obtained for recording. Interviews were continued until thematic saturation was reached, defined as no new codes or insights emerging in the final interviews. Code saturation was reached by Interview 22, and meaning saturation was confirmed by Interview 25 ^26^^,^^27^ (Supplementary File 3). The study was approved by the Ethics Committee of Alborz University of Medical Sciences (IR.ABZUMS.REC.1400.198). Transcripts were not returned to participants for comment.

Data from 25 conducted interviews were transcribed verbatim in Persian and analyzed using MAXQDA software. A thematic analysis method was employed to code the data.^28^ The analytic process reflected the six-phase framework described by Braun & Clarke^29^, which involves: (1) familiarization with the data, (2) generating initial codes, (3) searching for themes, (4) reviewing themes, (5) defining and naming themes, and (6) producing the report. Two researchers (JZ and FV) independently coded the transcripts. Coding discrepancies were resolved collaboratively, and the final coding framework was reviewed by senior qualitative researchers (ZS, NM). Peer debriefing sessions were held regularly to refine interpretations and ensure analytical rigor*.* Credibility was maintained through independent dual coding, peer debriefing with senior researchers, and maintenance of an audit trail.^30^ Coding of text segments resulted in relevant categories aligning with research objectives.

## 3 Results

A total of 25 community pharmacists participated in the study, comprising 15 males (60 %) and 10 females (40 %). The majority held a PharmD degree (22 participants), while 3 held a PhD. Participants were based in Tehran (16 pharmacists) and Alborz (9 pharmacists) provinces. Regarding professional background, 17 participants were pharmacist-owners, while 8 participants were employed pharmacists. Their work experience ranged from 4 years to over 18 years, with a median bracket of 10–14 years. Detailed participant characteristics are presented in Table 1.

**Table 1:** Participant characteristics.

Participant Code	Gender	Education	Province	Job Position	Work Experience (years)
P1	Male	PharmD	Tehran	Pharmacist+ Owner	4–10
P2	Female	PharmD	Alborz	Pharmacist+ Owner	4–10
P3	Male	PharmD	Tehran	Pharmacist+ Owner	>18
P4	Male	PharmD	Tehran	Pharmacist	14–18
P5	Female	PharmD	Alborz	Pharmacist+ Owner	10–14
P6	Male	PharmD	Tehran	Pharmacist	10–14
P7	Male	PharmD	Tehran	Pharmacist+ Owner	10–14
P8	Female	PhD	Alborz	Pharmacist+ Owner	>18
P9	Male	PharmD	Tehran	Pharmacist+ Owner	14–18
P10	Female	PharmD	Tehran	Pharmacist+ Owner	10–14
P11	Male	PharmD	Alborz	Pharmacist	4–10
P12	Male	PharmD	Tehran	Pharmacist+ Owner	10–14
P13	Female	PharmD	Alborz	Pharmacist+ Owner	>18
P14	Male	PharmD	Tehran	Pharmacist+ Owner	10–14
P15	Male	PharmD	Tehran	Pharmacist	>18
P16	Male	PharmD	Alborz	Pharmacist+ Owner	10–14
P17	Female	PhD	Tehran	Pharmacist	4–10
P18	Male	PharmD	Tehran	Pharmacist+ Owner	>18
P19	Male	PharmD	Tehran	Pharmacist+ Owner	14–18
P20	Female	PharmD	Alborz	Pharmacist	10–14
P21	Male	PharmD	Tehran	Pharmacist+ Owner	14–18
P22	Male	PharmD	Tehran	Pharmacist+ Owner	>18
P23	Female	PharmD	Alborz	Pharmacist+ Owner	10–14
P24	Male	PhD	Tehran	Pharmacist	4–10
P25	Female	PharmD	Alborz	Pharmacist+ Owner	10–14

From the 25 interviews, a total of 1260 initial codes and 11 main codes (categories) were extracted, reflecting the depth and richness of the data. The interviews collectively spanned 751 min, offering a comprehensive exploration of the experiences of pharmacies in managing the COVID-19 pandemic. Among the interviews, 23 were conducted in person, allowing for face-to-face interactions, while two were conducted via online messaging platforms. The varied modes of interviews added a nuanced perspective to the insights gathered.

**Fig. 1:**
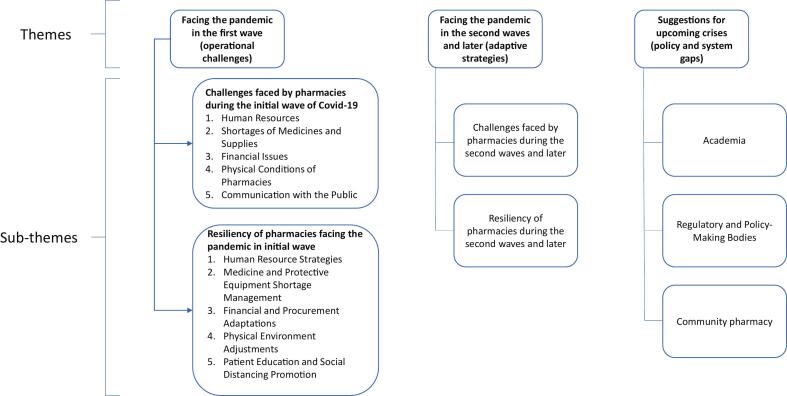
Thematic map.

The primary codes extracted from the interviews were organized into three themes, providing a structured framework for understanding the experiences of pharmacies in managing the COVID-19 pandemic: 1. Operational Challenges 2. Adaptive Strategies, and 3. Policy and System Gaps. Theme one, facing the pandemic in the first wave (tolerance of pharmacy in crisis) followed by two sub-themes outlining key challenges pharmacies faced early in the COVID-19 pandemic, and resiliency of pharmacies facing the pandemic in initial wave. Theme two, resiliency of pharmacies in the second waves and later pointing out the challenges and how pharmacies managed the COVID-19 pandemic, and theme three suggested solutions for future emergencies (later resiliency). A thematic map (Fig. 1) outlines the main themes and subthemes.

### 3.1 Qualitative results

#### 3.1.1 Theme one; facing the pandemic in the first wave (operational challenges)

##### 3.1.1.1 Sub-theme one; challenges faced by pharmacies during the initial wave of COVID-19

Amidst the onset of the COVID-19 pandemic, pharmacies grappled with a myriad of conditions that profoundly influenced their operations. These challenges included a surge in customer visits, shifts in consumer demands, uncertainty regarding treatment guidelines, the impact of medical facility closures, psychological strain experienced by both clients and staff, financial complexities, and obstacles in obtaining essential medications. These varied conditions played a pivotal role in shaping the challenges faced by pharmacies during the initial phase of the pandemic. The challenges identified by interviewees were categorized into five key areas.

1. Human Resources

Interviewees highlighted the psychological challenges faced by pharmacy staff during the pandemic, including fear, stress, and a lack of job security. The increased workload led to heightened nervousness and fatigue among personnel, making it more challenging for them to work while wearing masks and gloves.

*“Our stress levels increased a lot. Being constantly exposed to people and the virus—especially in the early days when so little was known—created a great deal of anxiety. We didn't know what would happen if we got infected ourselves; it felt as though we were constantly on the edge between life and death. All of this intensified the psychological pressure we were under.”* (P7).

Some interviewees reported a lack of time for rest, and responding to clients' requests for medicine and PPE became time-consuming. In their personal lives, both pharmacy staff and pharmacists also faced challenges. Their main concern was the risk of transmitting the disease to family members due to working in the potentially contaminated environment of the pharmacy. *“The pandemic had an undeniable impact on my personal life, especially because our workload increased so much during that time. Of course, everyone reacts differently, but I personally became more anxious and irritable than before. It definitely took a toll on my personal well-being.”* (P12).

Another key human resource challenges during the early phase of the pandemic were the shortage of staff and the unavailability of temporary personnel to support operations on particularly busy days: *“… Suddenly some of our staff became ill and we faced a shortage of pharmacists”.* (P5).

Interviewees highlighted two main challenges: *“extended working hours”* (P7) and *“increased workload”* (P18) for pharmacy staff, and “weakened immune systems due to heightened stress and fatigue.

2. Shortages of Medicines and Supplies

Some interviewees noted a one-day shortage of masks following the pandemic announcement. Additionally, pharmacies faced shortages of disinfectant gel, alcohol, drugs for COVID treatment, immune-boosting nutritional supplements and over-the-counter (OTC) medicines, elastic gloves, and oxygen capsules, adding to the challenges reported by pharmacists.

*“On Thursday, it (the pandemic) was announced. All our masks were completely sold out by Friday because of a sudden rush of customers. These included both filtered and regular masks, which hardly anyone had been using before. When I arrived at the pharmacy on Saturday, there wasn't a single mask left—not even for our own staff.”* (P3).

*“The medications introduced in the new protocols became a burden for pharmacies because everyone needed them. Due to shortages of these medications, people had to visit multiple pharmacies to find what they required”.* (P12).

Participants mentioned *“Disruptions and breakdowns in the supply chain for a large portion of the goods and medications”* (P21) a key challenge in meeting increased demands for medications and supplies and leading the items out of stock.

3. Financial Issues

The inability to predict demand *(“a state of ambiguity”*) (P1), fluctuations in medicine and PPE prices (“*highly erratic prices*”) (P14), and a lack of liquidity posed difficulties in providing medicines and managing staff. The market uncertainty, resulting from the COVID-19 pandemic, led some pharmaceutical companies to accept only cash transactions or compelled pharmacies to purchase a basket of medicines and PPE to ensure sales. Reduction of refill prescriptions and lack of adherence to treatment plans led to a decrease in pharmacies sales: *“Many pharmacies experienced a significant drop in sales because patients were no longer following up on their treatment plans. Some even faced financial difficulties. However, things gradually improved, as life simply couldn't come to a halt.”* (P9)

4. Physical Conditions of Pharmacies

The installation of glass or plastic barriers in pharmacies created challenges in sound transmission, prompting the use of loudspeakers to address communication issues.

*“The loudspeakers were all speaking at the same time, and their voices overlapped and created confusion”.* (P4).

*“Before COVID-19, we didn't have glass barriers around the counter, but they were installed afterward. As a result, sound didn't carry well through them”.* (P22)

5. Communication with the Public

Positive interactions between pharmacists and clients declined during the pandemic, while negative encounters and tensions became more frequent. Limited time for counseling made it increasingly difficult to educate the public or justify professional decisions. As one pharmacist noted,

*“It was challenging to explain to customers that I, as a pharmacist, could not dispense, for example, hydroxychloroquine due to its potential side effects”* (P6)*.*

The situation was further complicated by interference from individuals lacking relevant expertise: *“Those without expertise often interfered, which only added to the problems and created further difficulties for us.”* (P13).

##### 3.1.1.2 Sub-theme two; resiliency of pharmacies facing the pandemic in initial wave

Management of pharmacy conditions during the initial pandemic phase was categorized into five main areas based on the challenges faced:

1. Human Resource Strategies

Participants highlighted various strategies for managing human resources during the pandemic. These included adjusting staff duties, providing written descriptions of responsibilities, and dividing tasks based on staff members' stress levels related to COVID-19.

*“Since the sales pattern had been changed, I assigned one staff member to handle mask sales, another to sell hand sanitizers, and a third to manage alcohol sales, in order to prevent crowding.”* (P5).

Some pharmacies adjusted working hours and reduced shifts to alleviate workload pressure. Collaboration between different parts of the pharmacy was encouraged in understaffed establishments, and some pharmacies engaged in staff exchanges. Healthcare protocols, including the use of masks, gloves, and disinfectants, were enforced to protect employees. Protective practices, such as separating utensils, were implemented. Preparedness for staff infections involved scenario planning, symptom recognition, and offering support, both financial and medical. Testing for COVID-19 was mandated for all staff if one member was infected. Financial incentives, salary increases, loans, and pandemic-related training were provided to support staff:

*“We always considered that whenever the workload increased, a bonus would be allocated to help the staff better manage the additional pressure.”* (P25).

In personal life management, pharmacists adopted measures such as limiting communication, creating positive home environments, taking deputy positions, forming support groups, and sharing experiences.

2. Medicine and Protective Equipment Shortage Management

To address shortages of medicines and protective equipment, pharmacies adopted strategies such as limited and rationed distribution of rare supplies. Medicine bartering between pharmacies and restrictions on non-prescription medicine sales were used to prevent shortages. Repackaging disinfectants into smaller bottles and the use of alternative disinfectants were additional measures: *“We diluted 96%, 99%, or 70% alcohol into smaller bottles so we could hand them out to people and help manage the situation.”* (P2) Managing mask shortages involved purchasing from gray markets, providing tutorials on mask-making at home, and repackaging masks in smaller quantities: *“At times, we had to open a box of masks and repackage them into smaller plastic bags to ensure more people could receive them.”* (P10)

3. Financial and Procurement Adaptations

Purchasing and procurement management became a critical focus for pharmacists during the first wave of the pandemic. Pharmacies extended their medicine depots, converted capital into cash, and continuously monitored stock and essential supplies in real time as it was explained:

*“It's like building a dam and saying, ‘There's enough water stored behind it, so I can get through the summer—even if there's a drought.’*” (P3).

Compliance with COVID-19 treatment protocols and prescriptions was ensured to maintain access to necessary medications. Collaboration with small distributing companies aimed to enhance purchase alternatives and secure larger quotas, addressing financial challenges.

4. Physical Environment Adjustments

Pharmacies implemented various measures to manage their physical environment during the pandemic. Barriers, both plastic and glass, were installed around the counters. Positive air pressure systems were employed in some pharmacies, and air conditioning was adjusted for optimal airflow:

*“We tried to maintain positive air pressure in the staff area. As a result, throughout the entire COVID-19 period, we didn't have a single case of staff infection.”* (P18).

Doors and windows were kept open to maintain air circulation, even in cold seasons. Increased cleaning frequency and patient screening at the entrance based on prescriptions were implemented. Services for *COVID-19* patients were provided outside the pharmacy. Moreover, formal procedures were established:

*“We prepared a set of written guidelines and a checklist was marked every morning and evening to confirm that the pharmacy's hygiene protocols had been properly followed. This proved to be very helpful.”* (P15)

5. Patient Education and Social Distancing Promotion

Pharmacists implemented educational measures to enforce social distancing, including the use of posters and verbal advice to patients. Mandating masks for patients and providing them free of charge were common measures. Additional protective measures included placing alcohol disinfectant devices for patients. One participant explained,

*“We installed disinfectant dispensers for patients.”* (P15).

Pharmacists actively managed medication contraindications to reduce visits to physicians and hospitals. Telephone counseling, introducing reliable information sources to patients, promoting lifestyle modifications. As one participant noted:

*“…We had even prepared lists titled ‘immune-boosting medications’ as alternatives to antibiotics, to prevent weakening the immune system or contributing to antibiotic resistance.”* (P20).

Delivering medicines for patients with COVID-19 and chronic conditions were other health promotional measures, aiming to minimize in-person contact and reduce the risk of infection spread:

*“… if we recognized that a patient had* COVID-19*, we made sure to serve them as quickly as possible to reduce their contact with others. In some cases, we encouraged them to leave the pharmacy and arranged for their medications to be delivered to their home.”* (P23)

#### 3.1.2 Theme two; facing the pandemic in the second waves and later (adaptive strategies)

During the COVID-19 pandemic, pharmacies adapted their operations to prevent shortages of supplies like masks and alcohol sanitizer. Effective communication and purchasing strategies helped maintain stock through subsequent waves of infections. While the public initially feared the virus, these concerns diminished over time as pharmacy staff normalized workflows and adhered to safety protocols. Pharmacy operations observed fluctuations in medication dispensing patterns aligned with COVID-19 treatment recommendations. For example, demand rose for antivirals like Favipiravir and Remdesivir. Referrals for medical consultations also increased at pharmacies during this period. Disease transmission theories evolved as restrictions eased between waves. Compared to earlier phases, pharmacy staff demonstrated greater patience and tolerance when interfacing with the public amidst the uncertainties of the ongoing pandemic.

##### 3.1.2.1 Sub-theme one; challenges faced by pharmacies during the second waves and later

Through successive waves of the COVID-19 pandemic, pharmacies encountered multifaceted challenges in maintaining operations and services. Persistent and expanding drug shortages coupled with fluctuating monthly pharmaceutical sales volumes presented difficulties in inventory management, especially for medications used in COVID-19 treatment guidelines. Moreover, financial constraints emerged as pharmaceutical suppliers instituted new purchase terms including cash-only payments and bundling of non-COVID medications with high-demand COVID-19 therapeutics. The implementation of policy changes such as electronic prescribing programs and drug registration system updates further complicated pharmacy operations, as these changes coincided with peaks in COVID-19 cases.

*“The insurance system was updated, requiring pharmacy staff and pharmacists to undergo training. Ideally, this training should have taken place during quieter hours, but due to the heavy workload, it had to be done amid the busy pharmacy environment. Since many physicians were reluctant to fully adopt electronic prescriptions, pharmacies were forced to convert paper prescriptions into electronic formats themselves. This added a significant burden that could have been managed more effectively after the pandemic.”* (P11).

In summary, pharmacies faced significant resiliency challenges in the form of drug shortages, purchasing restrictions, policy shifts, and workforce constraints throughout successive waves of the dynamic COVID-19 pandemic environment.

##### 3.1.2.2 Sub-theme two; resiliency of pharmacies during the second waves and later

Pharmacy management during the second and subsequent waves is categorized into five categories: human resources management, procurement and supply chain management for PPE and medicines, heightened involvement in disease management, public education and promotional measures, and increased physical protection in pharmacies.

In terms of human resources management, rapid staff vaccinations and the implementation of rotating shifts in some pharmacies were undertaken, contributing to improved workload management.

Compared to the first wave, there was an enhanced proficiency in procuring medicines during crisis and shortage conditions. Pharmacies adopted more strategic approaches to purchasing, preventing shortages of protective equipment.

Pharmacies intensified their educational efforts regarding disease protection and the importance of vaccination. Additionally, the availability of protection and care items increased within pharmacies. As global awareness increased, pharmacies assumed a more active role in disease management. The objective was to reduce the burden on hospitals and physicians, emphasizing adherence to health protocols.

*“About a year after the onset of the pandemic, whenever we suspected someone had COVID-19, we tried to avoid referring them to physicians due to the high patient volume in hospitals. Instead, we made efforts to provide symptomatic treatment within the pharmacy itself.”* (P24).

#### 3.1.3 Theme three; suggestions for upcoming crises (policy and system gaps)

Suggestions provided by participants for managing future crises like the COVID-19 pandemic were classified into three main categories.

##### 3.1.3.1 Sub-theme one; academia

Participants recommended conducting studies to explore solutions that enhance the resilience of healthcare systems during crises. Additionally, they proposed investigating the role of media in professions involving direct contact with people. Suggestions for academia included training pharmacists on effective emergency management measures and incorporating COVID related topics into university courses.

*“Perhaps an evaluation is also needed regarding the role of authorities and higher-level organizations and what actions they could have taken. In my opinion, an even more important question is what they actually did to improve the situation, or conversely, what actions they took that they should not have. These two factors had a direct and significant impact on pharmacy operations.”* (P19)

##### 3.1.3.2 Sub-theme two; regulatory and policy-making bodies

Participants proposed several recommendations to improve pharmacy operations and resiliency in future pandemics. Developing and communicating guidelines for medicine and supply distribution in times of shortage was advised, along with enforcing physician adherence to such protocols to streamline pharmacy supply management.

*“… there should be a standardized prescription format overseen by the Ministry of Health, which would require physicians to write prescriptions based on scientific guidelines. It should not be left to individual discretion to prescribe any medication they wish.”* (P16).

Timely notification of disease onset would allow healthcare organizations more time to implement adaptive measures; for example, pharmaceutical companies could adjust production plans to meet market needs, while pharmacies could procure appropriate stocks of medications and supplies.

*“The drugs included in the treatment protocol should be communicated to the manufacturers at least two weeks in advance so they can increase their production.”* (P1).

Furthermore, earlier pandemic announcements could promote disaster readiness among the public and healthcare providers. Prioritizing pharmacy staff vaccination was emphasized, along with advocating for financial support from government agencies to mitigate pandemic-related revenue losses. Interviewees highlighted the need for policymakers to formally recognize the role of pharmacists in providing expanded medical services during pandemics.

*“If another pandemic occurs, it is the Ministry of Health's responsibility to provide us with training within a few days and to establish standard protocols in each area to ensure that proper procedures are followed*.” (P8).

Finally, they advised that pharmacists' experiences during the COVID-19 pandemic should be documented to inform future pandemic responses. In summary, participants proposed strategies focused on communication, supply chain resilience, public awareness, staff protection, financial stability, role recognition, and documenting lessons learned to improve pharmacy pandemic preparedness.

##### 3.1.3.3 Sub-theme three; community pharmacy

To improve pharmacy environmental safety during pandemics, participants recommended enhanced physical protections like barriers around service counters along with continuous air ventilation. Additional proposed measures included utilizing prescription delivery services and conducting transactions at the pharmacy entrance to limit in-store customer density.

*“The psychological pressure was so overwhelming that I wished I could have assigned someone to stand in an outdoor station to prevent people from coming inside and asking why we weren't giving them masks or hand sanitizers. Distributing these items outside would have helped reduce the stress and tension they were causing for the staff.*” (P8).

A participant suggested ticketing machine:

*“… the ticketing machine had a significant impact. Before we installed it, the space in front of the counter—intended for patients—was overcrowded. Even someone who had already received their medication couldn't leave easily; they had to push through the crowd. But this system helped us greatly by making the flow of people more organized and reducing congestion.”* (P14).

For human resource management, suggestions focused on refined task coordination and expanded staffing to distribute workloads, which could help reduce employee stress and disease transmission risks*:*

*“… it's clear that in such situations, regardless of how many employees you already have, it may be necessary to increase the workforce and reduce working hours in order to prevent burnout and minimize the risk of errors.”* (P21).

Building mutual understanding among staff and providing financial support were also advised to support pharmacy teams during crises.

To address financial challenges, interviewees advocated for rational purchasing of pharmaceuticals during pandemics and avoiding *“*impulsive buying*”* (P17). They also emphasized the need to ensure stockpiles of medications and equipment, as well as equitable distribution of resources among people to strengthen pharmacy resilience throughout outbreaks.

In summary, the proposed strategies highlighted optimizing pharmacy layouts, workflows, staffing, and inventory management practices to enhance operations and safety during pandemic events.

## 4 Discussion

Our qualitative study explored how community pharmacists in Tehran and Alborz provinces in Iran experienced and responded to the challenges of the COVID-19 pandemic. The findings revealed three overarching themes: operational challenges, adaptive strategies, and policy and system gaps. Together, these results demonstrate both the resilience of community pharmacists and the systemic vulnerabilities in Iran that must be addressed to improve healthcare preparedness for future crises. Our findings mirror reports from other countries where community pharmacists served as the first point of contact for patients,^31^ yet faced key challenges encountered in emergency management including human resources, medicine shortages, and financial issues. 32, 33, 34, 35

In addressing human resource challenges, a notable concern was the psychological well-being of pharmacy personnel. Increased workload, extended working hours, staff infections, and limited labor availability were identified as critical factors. Studies conducted in the United States and Canada emphasized the importance of psychological well-being among pharmacy personnel during the pandemic.^36^^,^^37^ Pharmacies implemented various measures to address these challenges, such as adopting a single-task approach, dividing tasks, reducing shift hours, and incorporating short breaks to enhance staff resilience.^4^^,^^34^ Pharmacies that operated for more than 8 h a day or handled a high volume of customers were more likely to provide inaccurate information due to factors such as extended working hours, fatigue, and anxiety. This issue could have been managed by ensuring sufficient staffing and appropriately distributing responsibilities among employees.^38^ A study on the resilience of pharmacies during COVID-19 in the UK concluded that flexible opening hour was advantageous for completing essential tasks such as prescription processing, monitoring the effectiveness of interventions, and disinfecting workspaces, while also fostering staff wellbeing.^33^ Esmaeili et al.^39^ quantified pharmacy preparedness levels of emergency in Tehran using a five-dimensional model. According to the study, pharmacies had the highest level of preparedness in physical facilities, 52 % in software, 41.7 % in medical consumables, 32.7 % in human resources training and management, and 27.6 % in medicines. As physical preparedness indicators, this model included multiple entrance doors in pharmacies, construction and non-construction resistance, emergency power availability, and dry ice availability.^39^ Our findings suggest efficient management of pharmacies' physical condition played a crucial role in alleviating staff stress and enhancing protection. Common practices included installing barriers around the counter to minimize direct contact, ensuring a safe distance, spatial rearrangement, and setting up queues. Nevertheless, such physical distance measures were not possible due to lack of space in some pharmacies.^32^^,^^33^ Some pharmacies also implemented strategies like separating prescriptions of COVID-19 patients from others at the front door, reducing disease transmission.^32^ Although, it was indicated that pharmacists viewed this measure as potentially increasing the risk of infection for pharmacy staff, as it could encourage individuals to visit the pharmacy, which they might perceive as a safe environment. Additionally, it was reported that implementing this measure would be impractical for community pharmacies due to the associated costs and time requirements.^6^ These measures could be integrated into pharmacy audit checklists by regulatory bodies to ensure proper physical conditions during crises. In this study, a significant hurdle faced by pharmacies involved shortages of essential medicines and PPE during the initial stages of the epidemic. Previous studies have highlighted shortages in community pharmacies.^40^, ^41^, ^42^ To address these shortages, pharmacies adopted strategies such as rationing scarce medicines. Bahlol^32^ mentioned rationing medicines like paracetamol or Hydroxychloroquine to serve a larger number of patients in Egypt. The effort of steady supply contributed to the creation of a “pharmacy emergency support guarantee system” in China, which focused on addressing drug shortages through mechanisms like active surveillance, early alerts, and monitoring the safety of prescribed medications.^5^ Additionally, Bragazzi et al.^5^ highlighted the importance of emerging technologies in maintaining the supply of pharmacies. Pharmacists and healthcare providers are encouraged to leverage digital tools powered by Big Data and Artificial Intelligence (AI) to enhance real-time monitoring and respond to healthcare utilization trends. The correlation between web search volumes and prescription patterns enables pharmacists to track digital interest in disease-related medications and improve pharmaceutical care and logistics during the pandemic.^5^ During the COVID-19 pandemic, health systems adopted two primary strategies for PPEs and ventilator procurement: responsiveness and preparedness. Responsiveness strategies address immediate needs as the crisis occurs, with decision-makers enhancing domestic production, supporting innovative supply methods, borrowing resources from the private sector, and competing in the global market. In contrast, preparedness strategies, such as stockpiling, emphasized on long-term solutions prior to a crisis. Many health systems implemented these strategies during the COVID-19 pandemic.^14^ By implementing the preparedness strategy, community pharmacies in Malaysia had adequate supplies of PPE to manage the emergency.^35^ The result of this study demonstrated the importance of formulating guidelines for the distribution of medicines and PPEs during shortages, along with comprehensive treatment guidelines. Government-led initiatives providing professional guidelines for managing epidemic conditions were highlighted as essential for pharmacists' preparedness and response. Lessons from China's response to the pandemic, where the outbreak first emerged, highlight the significance of comprehensive guidelines for managing public health crises. The Chinese Pharmaceutical Association (CPA) published expert consensus documents on hospital and retail pharmacy strategies for handling COVID-19. Building on these, the International Pharmaceutical Federation (FIP), in collaboration with the CPA, developed a guidance that outlines essential preventive measures, recommended equipment, pharmacist advice, and laboratory testing protocols.^43^ Austin and Gregory,^34^ in 2021 in Ontario, Canada, emphasized the government's role in providing professional and integrated guidelines for managing epidemic conditions. Proposing instructions through official channels and clarifying pharmacists' roles in various situations had a positive impact on their preparedness and response. Our finding aligns with research from other regions illustrating that pharmacists' frontline roles are often undervalued within formal emergency frameworks. For example, in Saudi Arabia, pharmacists possess competencies across prevention, preparedness, response, and recovery phases, yet systemic barriers limit their formal inclusion in disaster planning.^44^ Integrating pharmacists into national preparedness strategies is thus critical to optimize resource allocation and bolster system resilience during future crises. Consistent with the findings of this study, Communication challenges arose early in the COVID-19 pandemic, diminishing positive interactions between pharmacists and the public and often resulting in negative encounters. As noted in Zaidi and Hasan's 2021 study based in United Kingdom,^40^ factors such as drug shortages contributed to unfavorable public perceptions of pharmacies during the pandemic. Their findings and the results of this study highlight the need to educate and empower pharmacists to manage patient interactions effectively during times of social and health crises. Doing so can help overcome communication barriers and maintain constructive provider-patient relationships in future pandemic scenarios. Proactive training focused on communication crisis, managing conflicts, and restoring trust may prove beneficial for improving pharmacist-public interactions when drug supply constraints and other stressors emerge. Despite these constraints, pharmacists demonstrated remarkable adaptability by innovating service delivery and embracing technology. Pharmacies implemented various educational and promotional measures during the COVID-19 pandemic to encourage public health practices. Such efforts included teleconsultations, displaying informational posters, implementing strict safety measures including encouraging wearing of masks, and providing hand sanitizer to promote adherence to disease prevention protocols. As noted in previous studies, pharmacies also played a role in mandating social distancing within stores.^32^, ^33^, ^34^ In terms of direct public education, pharmacists offered in-person and telephone consultations to provide advice about COVID-19. During the COVID-19 pandemic, telehealth and tele-pharmacy services, including patient counseling and drug information provided through telecommunication technologies, were promoted as they offered enhanced protection for both patients and healthcare providers.^45^^,^^46^ These services aimed to reduce unnecessary physician visits and healthcare facility workloads during the pandemic. Many studies highlighted the critical role of pharmacy-based patient education and consulting during the COVID-19 outbreak via telephone and mobile applications.^31^^,^^33^^,^^43^^,^^47^ This underscores the importance of institutionalizing telepharmacy and integrating digital health platforms into pharmacy practice to enhance responsiveness during emergencies. An important role of pharmacists during the pandemic was the early identification of patients, which helped alleviate the burden on hospital staff and physicians in other countries.^32^^,^^35^^,^^47^^,^^48^ Our findings suggest that pharmacists contributed indirectly to the early identification of suspected COVID-19 cases by educating patients about signs and symptoms. However, community pharmacies in Iran did not play a systematic role in case identification, which may be attributed to the heavy workload and resource constraints experienced during the pandemic.^49^ This critical role was not identified in this research. Although this study focused on the experiences of community pharmacists during the COVID-19 pandemic, our findings carry broader lessons that extend well beyond this single event. The pandemic highlighted the critical role of community pharmacies as accessible healthcare providers, yet it also revealed systemic weaknesses that limited their potential contribution. Integrating pharmacists more fully into national disaster and pandemic preparedness frameworks would ensure that their expertise and accessibility are leveraged during future public health emergencies. This study also underscores the importance of investing in telepharmacy infrastructure to facilitate remote consultations, medication delivery, and continuity of care when physical access is disrupted.^50^ Furthermore, strengthening pharmaceutical supply chains is vital to avoid critical shortages of medicines and protective equipment during emergencies. Finally, the experiences shared by participants highlight the need for psychosocial and financial support mechanisms to safeguard the well-being of pharmacists, who often shoulder intense workloads and emotional strain in times of crisis. By adopting these measures, health systems can position community pharmacies as strategic partners, enhancing overall resilience and improving the capacity to respond effectively to future pandemics and other public health emergencies.

### 4.1 Limitations

This study has some limitations. Recall bias is a concern given the time elapsed between the early pandemic waves and data collection, which may have affected participants' recollections of events. Reporting bias is another potential issue, where interviewees may respond based on perceived desired answers rather than factual recall. Additionally, as the data reflects experiences within a specific region, caution should be exercised when generalizing the findings to other geographic areas or contexts. Further research across broader samples could build upon these findings to develop a more comprehensive understanding of pharmacy experiences throughout the COVID-19 pandemic. However, within the scope of this study, the potential for recall and reporting biases should be considered when interpreting the results.

## 5 Conclusions

This study elucidates the experiences of community pharmacies in Iran throughout the COVID-19 pandemic through the lens of resilience. The findings offer practical insights for pharmacy stakeholders including owners, healthcare policymakers, professional associations, and researchers regarding crisis management and adaptation strategies for widespread outbreaks. Key challenges emerging in the initial pandemic wave encompassed human resource constraints and shortages of medicines and PPE. Pharmacies responded by refining task coordination, providing staff support, rationalizing supplies, and adopting strategic purchasing protocols. In subsequent waves, pharmacies demonstrated enhanced resiliency and took on expanded roles in public health education, disease management, and reducing care delivery burdens on hospitals and physicians. However, the critical contributions of community pharmacies in outbreak response have yet to be adequately incorporated into policy frameworks. In light of these findings, policymakers should prioritize integrating community pharmacies into formal pandemic preparedness and response plans, strengthen pharmaceutical supply chain resilience, and invest in telepharmacy infrastructure to ensure continuity of care during emergencies, with the goal of optimizing future emergency preparedness and response. The COVID-19 pandemic underscores the importance of recognizing community pharmacies' roles in health education, disease mitigation, transmission control, and healthcare workload distribution during public health emergencies. Future research should further explore effective strategies to institutionalize community pharmacy involvement in emergency management and evaluate innovative digital health approaches, such as telepharmacy to strengthen health system resilience.

## CRediT authorship contribution statement

**Zhivan Zomorodi:** Writing – original draft, Project administration, Data curation. **Faezeh Valaei Sharif:** Writing – review & editing, Conceptualization, Project administration. **Najmeh Moradi:** Writing – review & editing, Formal analysis. **Zahra Sharif:** Supervision, Methodology, Conceptualization.

## Funding

This research did not receive any specific grant from funding agencies in the public, commercial, or not-for-profit sectors.

## Declaration of competing interest

The authors declare none.

## Appendix A Supplementary data

Supplementary material 1 Image 1

Supplementary material 2 Image 2

Supplementary material 3 Image 3
