# Supplementary material for: Community pharmacy resiliency during Covid-19 pandemic in Iran: A qualitative study
Source: Explor Res Clin Soc Pharm. 2025 Oct 3;20:100670. doi: 10.1016/j.rcsop.2025.100670 (PMC12553000; doi:10.1016/j.rcsop.2025.100670)
Supplement: Supplementary file 1 — Supplementary material 1 [file mmc1.docx]

Supplementary File

| Interview Number | Cumulative Codes Identified | New Codes per Interview | % of Total Codes |
| --- | --- | --- | --- |
| 1 | 79 | 79 | 6.27% |
| 2 | 151 | 72 | 5.71% |
| 3 | 227 | 76 | 6.03% |
| 4 | 307 | 80 | 6.35% |
| 5 | 389 | 82 | 6.51% |
| 6 | 456 | 67 | 5.32% |
| 7 | 527 | 71 | 5.63% |
| 8 | 602 | 75 | 5.95% |
| 9 | 674 | 72 | 5.71% |
| 10 | 756 | 82 | 6.51% |
| 11 | 774 | 18 | 1.43% |
| 12 | 797 | 23 | 1.83% |
| 13 | 818 | 21 | 1.67% |
| 14 | 838 | 20 | 1.59% |
| 15 | 860 | 22 | 1.75% |
| 16 | 873 | 13 | 1.03% |
| 17 | 895 | 22 | 1.75% |
| 18 | 911 | 16 | 1.27% |
| 19 | 930 | 19 | 1.51% |
| 20 | 952 | 22 | 1.75% |
| 21 | 956 | 4 | 0.32% |
| 22 | 960 | 4 | 0.32% |
| 23 | 960 | 0 | 0.00% |
| 24 | 961 | 1 | 0.08% |
| 25 | 961 | 0 | 0.00% |
