# Supplementary material for: Community pharmacy resiliency during Covid-19 pandemic in Iran: A qualitative study
Source: Explor Res Clin Soc Pharm. 2025 Oct 3;20:100670. doi: 10.1016/j.rcsop.2025.100670 (PMC12553000; doi:10.1016/j.rcsop.2025.100670)
Supplement: Supplementary file 3 — Supplementary material 3 [file mmc3.docx]

**Interview predefined questions**

| No | Questions | Interviewee guidance |
| --- | --- | --- |
| 1 | Ice breaking question:  How was your experience during COVID-19 in the pharmacy? | Seek for tolerability of pharmacies during initial weeks and the first reaction of pharmacy to pandemic |
| 2 | How did pharmacy routine activities and pharmaceutical services change after COVID-19? | Seek for changing in activities, their reaction during initial weeks, and pharmacy adaptability |
| 3 | How did you manage the shortages that pharmacy faced? Alcohol, face masks, … | Seek for tolerability and adaptability |
| 4 | How did you manage emotional pressures of the initial weeks? | Seek for emotional tolerability |
| 5 | How did you adapt the Physical conditions of pharmacies and manage staffs relations? | Seek for their reaction to infected staffs and their protective actions |
| 6 | What would you suggest for further crisis in future? What will you do for your pharmacy? |  |
